# Absolute Quantification of Isoflavones in the Flowers of *Pueraria lobata* by qHNMR

**DOI:** 10.3390/plants11040548

**Published:** 2022-02-18

**Authors:** Punam Thapa, Hye Mi Kim, Joon-Pyo Hong, Ranhee Kim, Sunil Babu Paudel, Hyukjae Choi, Dae Sik Jang, Joo-Won Nam

**Affiliations:** 1 College of Pharmacy, Yeungnam University, Gyeongsan-si 38541, Korea; pansup35@gmail.com (P.T.); phrsunil@gmail.com (S.B.P.); h5choi@yu.ac.kr (H.C.); 2 College of Pharmacy, Kyung Hee University, Seoul 02447, Korea; hyemi586@gmail.com; 3 Department of Life and Nanopharmaceutical Sciences, Graduate School, Kyung Hee University, Seoul 02447, Korea; ongjoon@naver.com (J.-P.H.); rhee0423@khu.ac.kr (R.K.); 4 Research Institute of Cell Culture, Yeungnam University, Gyeongsan-si 38541, Korea

**Keywords:** *Pueraria lobata*, Puerariae Flos, isoflavone, quantitative proton nuclear magnetic resonance (qHNMR), high-performance liquid chromatography (HPLC)

## Abstract

*Pueraria lobata* (Willd.) Ohwi. is a widely used medicinal plant in Korea, China, and Japan. The flower of *P. lobata* (Puerariae Flos) contains various bioactive substances such as triterpenoidal saponins and isoflavonoids. In this study, we developed a quantitative analysis of the isoflavones of Puerariae Flos by quantitative proton nuclear magnetic resonance (qHNMR) spectroscopy using the internal calibrant (IC). From the qHNMR results, the isoflavone content was found to be 7.99% and 10.57% for the MeOH sonication extract (PLs) and the MeOH reflux extract (PLr) of Puerariae Flos, respectively. The quantified isoflavone content was validated using the conventional analytical method, high-performance liquid chromatography with ultraviolet detection (HPLC-UV). The present study shows that validated qHNMR spectroscopy is a reliable method for quantifying and standardizing the isoflavone content in Puerariae Flos.

## 1. Introduction

*Pueraria lobata* (Willd.) Ohwi. is a perennial plant belonging to the Leguminosae family [1]. The high medicinal and nutritional value of the root of *P. lobata* (Puerariae Radix) has proven to be responsible for its traditional use as an ingredient in soups and Chinese cuisines [2,3]. Puerariae Radix contains isoflavonoids [4,5] and triterpenoidal saponins [6], which show estrogenic [4], antidipsotropic [7], antioxidant [5,8], antimutagenic [5], and antihepatotoxic activities [6]. Recently, there has been scientific interest in the flowers of *P. lobata* (Puerariae Flos) owing to the presence of isoflavonoids as the major constituents [1,9,10]. In addition to the roots and flowers, isoflavonoids were also reported from different parts of *P. lobata* such as outer barks [11], stems [12], and leaves [13]. Isoflavonoids are a large group of secondary metabolites in plants and include isoflavanones, isoflavans, rotenoids, and pterocarpans, which consist of two benzene rings linked by a three-carbon bridge that produces a 3-phenylchromen-4-one backbone [14,15].

Quantitative and qualitative information about natural products is important for ensuring the chemical consistency, efficacy, and safety of the materials [16]. Isoflavone components such as puerarin, genistein, kakkalide, irisolidone, daidzin, tectorigenin (**2**), and tectoridin (**5**) [1], are responsible for most of the pharmacological activities of Puerariae Flos such as antioxidant [1], antitumor [2], and antistroke [17] properties. Thus, identification and quantification of isoflavones are crucial for the quality control of the sources. Various analytical techniques have been developed to quantify isoflavones, such as high-performance liquid chromatography-photodiode array (HPLC-PDA), liquid chromatography-electrospray ionization-tandem mass spectrometry (LC-ESI-MS) [18], liquid chromatography coupled with triple stage quadrupole tandem mass spectrometry (TSQ-MS/MS) [19], ultra-performance liquid chromatography-electrospray ionization-tandem mass spectroscopy (UPLC-ESI-MS/MS) [20], and quantitative ^1^H NMR (qHNMR) [14,21].

**Figure 1:**
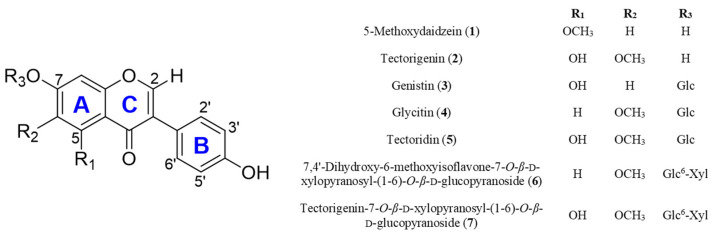
Chemical structures of isoflavones from Puerariae Flos.

qHNMR is a powerful analytical tool for purity determination and quantification of compounds in mixtures. There is a growing interest in the application of qHNMR methods to quantify various classes of components in natural products [22,23]. qHNMR is preferable over conventional chromatography-based quantitative methods because it allows for the simultaneous identification and quantification of the complex matrix in a single run. The integration of an individual resonance from different chemical markers depends on the number of protons and the molar concentration of the compound [22]. There are four methods for performing quantitative analysis: (i) relative 100% method without calibration, (ii) internal calibration (IC), (iii) external calibration (EC), and (iv) external calibration of the internal solvent signal (ECIC) [24]. Among them, IC is a direct method for the simultaneous measurement of standard and sample mixtures dissolved in solvents. IC does not require a calibration curve with an external calibrant, and it is the most common and easiest approach for absolute quantitation [24]. Because the samples and calibrants were subjected to identical experimental parameters, the analytical error is significantly smaller than that in other methods and the experimental time is shortened [22]. The objective of the present study was to develop a validated qHNMR method using proton signal selection criteria to accurately determine the isoflavone content in two extracts of Puerariae Flos: 5-methoxydaidzein (**1**) [25], tectorigenin (**2**) [1], genistin (**3**) [26], glycitin (**4**) [1], tectoridin (**5**) [1], 7,4′-dihydroxy-6-methoxyisoflavone-7-*O*-*β*-d-xylopyranosyl-(1-6)-*O*-*β*-d-glucopyranoside (**6**) [27], and tectorigenin-7-*O*-*β*-d-xylopyranosyl-(1-6)-*O*-*β*-d-glucopyranoside (**7**) [1] (Figure 1). The quantitative results obtained by qHNMR were verified using conventional chromatography. The present method can be applied to the standardization and chemical profiling of the raw materials of dietary supplements and foods using Puerariae Flos.

## 2. Results and Discussion

### 2.1. ^1^H NMR Signal Assignment and Identification

**Figure 2:**
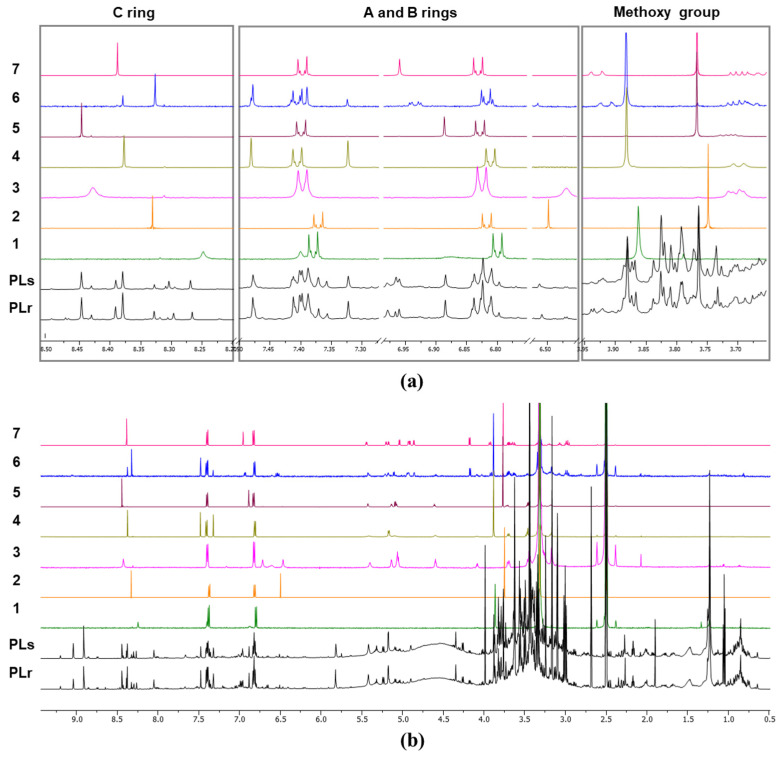
qHNMR spectra of PLs (MeOH sonication), PLr (MeOH reflux) extracts, and seven major isoflavones **1**–**7:** (**a**) expanded and (**b**) full qHNMR spectra.

The resonances for seven major isoflavones (**1**–**7**) of the Puerariae Flos extracts prepared by two different methods [MeOH sonication (PLs) and MeOH reflux (PLr)] were classified into three parts, as shown in Figure 2: (i) signals for the methoxy protons of the A-ring at 3.7–3.9 ppm, (ii) signals for an AA′XX′ spin system of the B ring and the H5/H6/H8 protons of the A ring at 6.3–7.5 ppm, (iii) H-2 singlets of the C-ring at 8.2–8.5 ppm. The first two areas (i and ii) showed intense signal overlaps, which can make precise signal assignment challenging and lead to inaccurate quantitation. However, relatively simple and well-separated singlets from the H-2 proton of the C-ring at 8.2–8.5 ppm are appropriate to assign and quantify the isoflavones in the extracts [14,21].

**Figure 3:**
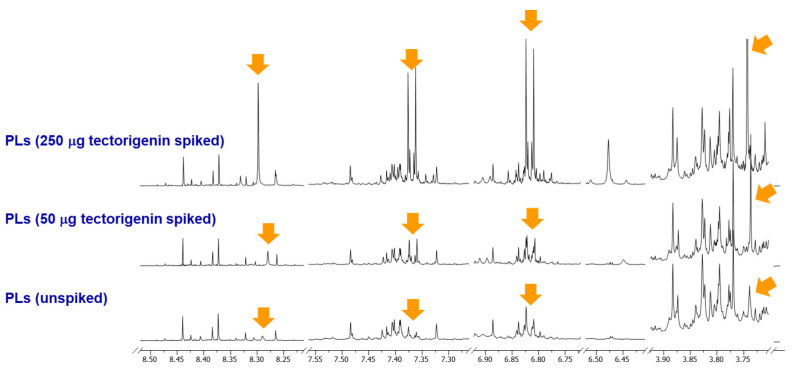
Stacked ^1^H NMR spectra of the unspiked PLs (MeOH sonication), 50 μg tectorigenin (**2**) spiked, and 250 μg tectorigenin (**2**) spiked samples.

The structures of isoflavones (**1**–**7**) have been identified as 5-methoxydaidzein (**1**) [25], tectorigenin (**2**) [1], genistin (**3**) [26], glycitin (**4**) [1], tectoridin (**5**) [1], 7,4′-dihydroxy-6-methoxyisoflavone 7-*O*-*β*-d-xylopyranosyl-(1-6)-*O*-*β*-d-glucopyranoside (**6**) [27], and tectorigenin-7-*O*-*β*-d-xylopyranosyl-(1-6)-*O*-*β*-d-glucopyranoside (**7**) [1] by analysis of the ^1^H NMR and low-resolution electrospray ionization-tandem mass spectrometry (LR-ESI-MS) data and comparison with reported values. The H-2 signal of compound **2** was found to migrate in the ^1^H NMR spectrum of the extract, and the shifted H-2 signal was confirmed by spiking experiments with pure standard tectorigenin (**2**) in the PLs extract as shown in Figure 3. This chemical shift inconsistency of the purified compound and the mixture state is caused by differences in the chemical environments, such as temperature, sample matrix, concentration, and pH.

### 2.2. Major Isoflavone Content Calculated by qHNMR

**Figure 4:**
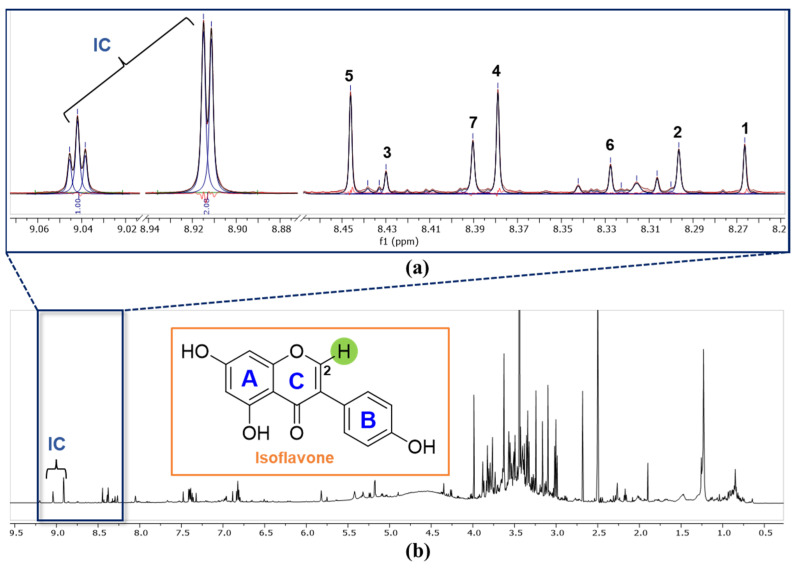
(**a**) Expansion of the resonances for H-2 and IC: The region for the deconvoluted signals of H-2 of isoflavones **1**–**7** in the extracts. Signals for internal calibrant (IC: methyl 3,5-dinitrobenzoate) and H-2 of **1**–**7** are labeled. The black, blue, and red-colored lines represent the peak sum, peak curves, and peak residual, respectively. (**b**) Full ^1^H NMR spectrum of PLs (MeOH sonication).

In the general qHNMR method, the relative and absolute contents of the target compounds were calculated using the peak integration method [28]. Because natural products are a mixture of various classes of compounds, when analyzing the NMR spectrum of the extract, it is difficult to separate and obtain the integration value of each peak due to signal overlapping. The precision and accuracy of qHNMR analysis depend on the accuracy of the integration of the target peak. In this study, although the resonances for H-2 of isoflavones were singlets and relatively well separated, the peak deconvolution method was applied to minimize the error in calculating the integral value. A global spectral deconvolution (GSD) function of MestReNova software was used to acquire peak integration. GSD is a fast and automatic algorithm that detects and deconvolutes all selected spectral peaks in a spectrum and extracts all impurities, noise, spikes, and signals from other untargeted molecules [29]. Methyl 3,5-dinitrobenzoate was selected as an internal calibrant for absolute quantitation, considering its stability and solubility [21]. A general criterion for selecting an internal calibrant is that it should be inactive to the sample, be of high purity, and show minimal signal overlap with the quantified signal [24]. The IC shows characteristic and well-defined signals at δ_H_ 8.91 (d, *J* = 2.2 Hz, 2H) and 9.04 (t, *J* = 2.2 Hz, 1H) ppm which were not overlapped with the constituent signals for the PLs and PLr extracts. Peak deconvolution was applied to the entire spectrum and quantified the H-2 singlet region at 8.2–8.5 ppm, resulting in black, blue, and red-colored lines representing the peak sum, peak curves, and peak residual, respectively. (Figure 4a). The chemical shifts at 8.45, 8.43, 8.39, 8.38, 8.33, 8.30, and 8.27 ppm were used to generate the areas and quantify the contents of compounds **5**, **3**, **7**, **4**, **6**, **2**, and **1**, respectively. The areas for each deconvoluted peak were determined using the GSD option with five fitting cycles. The absolute content of the target compounds was calculated using the IC method [24]. The calculated content % *w*/*w* of the seven isoflavones (**1**–**7**) in the PLs extract were 0.95, 0.67, 0.43, 1.96, 1.86, 0.57, and 1.55, respectively (Table 1). All the quantified amounts were above the amount calculated by the limit of quantitation (LOQ) performed for system validation.

### 2.3. Validation Studies

The validation results proved that the developed method is suitable for quantification. The ^1^H NMR method was validated in terms of sensitivity and accuracy based on the ICH guidelines [30].

### 2.4. Sensitivity

According to the ICH guidelines, the limit of detection (LOD) and the limit of quantitation (LOQ) of a liquid chromatography system are determined by calculating the signal-to-noise (S/N) ratio. The acceptable S/N ratios were 3:1 and 10:1 for the LOD and LOQ, respectively. In this experiment, the sensitivity parameter of the ^1^H NMR quantification method was determined by calculating the S/N ratio using the S/N ratio calculator function of the MestReNova software. The calibration curve was plotted based on linear regression analysis of the S/N ratio versus sample concentration, and the LOD (0.0014 mg in 600 μL) and LOQ (0.0036 mg in 600 μL) were calculated using a linear equation (Figure S15).

### 2.5. Accuracy

The accuracy parameter was determined using the spike-recovery method [21]. Three replicates of PLs containing a known amount of methyl 3,5-dinitrobenzoate were quantified using the GSD method before and after spiking with tectoridin (**5**). The sample was prepared as mentioned in the Experimental section. 50 μL of the stock solution containing 2500 μg in 200 μL of DMSO-*d_6_* was spiked into the three samples. The average recovery % of spiked **5** was 98.4072%, which was within the acceptable range (Figures S16 and S17) [31] and the relative error was 0.7868%. The recovery % was calculated using the following Equation (1):

(1) $$\mathrm{Recovery}\;\%=\frac{\mathrm{conc}.\;\mathrm{of}\;\left(\mathrm{spiked}\;\mathrm{sample}\;-\;\mathrm{unspiked}\;\mathrm{sample}\right)}{\mathrm{spiking}\;\mathrm{concentration}}\times 100$$

### 2.6. HPLC-UV Quantification Analysis

To compare the qHNMR results with other experimental methods, HPLC-UV-based quantification analysis was performed on three selected compounds, **2**, **5**, and **7**. Based on the retention time of the purified standard compounds (**2**, **5**, and **7**), the analytes in the PLs and PLr extracts were identified (Figure S18). Quantification of the isoflavones of the PLs and PLr was performed using a calibration curve for six concentrations of tectorigenin (**2**, 99.74% pure), tectoridin (**5**, 92.52% pure), and tectorigenin-7-*O*-*β*-d-xylopyranosyl-(1-6)-*O*-*β*-d-glucopyranoside (**7**, 99.33% pure) (Figure S18). The calculated quantities of compounds **2**, **5**, and **7** in the PLs and PLr are listed in Table 1.

### 2.7. Comparison of Two Different Sample Preparation Methods, PLr and PLs

Two different extraction methods were also explored: sonication and heat reflux. Sonication is a cavitation process that causes swelling and diffusion across the cell wall upon the application of sound energy, whereas heat reflux is accomplished by allowing hot solvent in solid tissue and leaching out the compounds. The extraction method is a crucial factor for varying the compositions and contents of the chemical compounds in an extract. Heat reflux was found to be a superior extraction method over the ultrasonic-assisted method because it gives high yields of bioactive chemical contents of polysaccharides, polyphenols, and flavonoids [32]. Similarly, as shown in the results (Table 1), the isoflavonoid content was higher in the MeOH reflux (PLr) extract than in the MeOH sonication (PLs) extract, indicating that the heat application method is better than the sound application method. 

## 3. Materials and Methods

### 3.1. General Experimental Procedures

The semi-preparative HPLC for the isolation was performed using a Waters system (Waters Corporation, Milford, CT, USA) equipped with a YMC-Pack ODS-AQ (10 × 250 mm, 5 μm) column (YMC Co., Ltd., Kyoto, Japan) and an Agilent C_18_-column (4.6 × 250 mm, 5 μm) (Agilent Technologies, Santa Clara, CA, USA). LR-ESI-MS was performed using an Agilent 6120 series LC-MS system (Agilent Technologies, Santa Clara, CA, USA).

### 3.2. Chemicals and Reagents

Methyl 3,5-dinitrobenzoate and DMSO-*d*_6_ were purchased from Sigma Aldrich (St. Louis, MO, USA) and Cambridge Isotope Laboratories (Andover, MA, USA), respectively. HPLC-grade acetonitrile was obtained from Fisher Scientific Korea Ltd. (Gangnam-gu, Seoul, Korea).

### 3.3. Plant Materials

The dried flowers of *P. lobata* (Puerariae Flos) were purchased from CK Pharm Co. Ltd., Seoul, Republic of Korea, in August 2019. The plant material was authenticated by one of the authors, Prof. Dae Sik Jang, and a voucher specimen (PULO4-2019) was deposited at the Laboratory of Natural Product Medicine, Kyung Hee University.

### 3.4. Extraction and Isolation

#### 3.4.1. MeOH Sonication of Puerariae Flos (PLs)

The commercially dried flowers of *P. lobata* without pulverization (20 g) were extracted with MeOH (200 mL × 2) by sonication in an ultrasonic bath (frequency of 40 kHz; Power Sonic 520, Hwashin Co., Daegu, Korea) at room temperature for 2 h. The combined extracts were filtered and then dried with a rotary vacuum evaporator (EYELA SB-1200, EYELA Tokyo Rikakikai Co., Ltd., Tokyo, Japan) at 40 °C to yield a MeOH extract (PLs, 1.44 g).

#### 3.4.2. MeOH Reflux of Puerariae Flos (PLr)

The same plant material as PLs (50 g) was extracted with MeOH (500 mL × 2) under reflux for 2 h. The solvent was removed using a rotary vacuum evaporator (EYELA SB-1200) at 40 °C to yield a MeOH extract (PLr, 7.9 g).

#### 3.4.3. Isolation of Compounds **1**–**7**

Tectorigenin (**2**, 57 mg), tectoridin (**5**, 247 mg), and tectorgenin-7-*O*-*β*-d-xylosylglucoside (**7**, 149 mg) were isolated from our previous study [33]. PLs (100 mg) was subjected to semi-preparative reversed-phase HPLC with an isocratic solvent system of CH_3_CN/H_2_O (20:80, 2 mL/min) to afford glycitin (**4**, 1.95 mg) and 7,4′-dihydroxy-6-methoxyisoflavone 7-*O*-*β*-d-xylopyranosyl-(1-6)-*O*-*β*-d-glucopyranoside (**6**, 0.51 mg). 5-Methoxydaidzein (**1**, 0.75 mg) and genistin (**3**, 0.43 mg) were isolated from the washing fraction (12.1 mg), which was collected while isolating **4** and **6**, using an isocratic solvent system of CH_3_CN/H_2_O (40:60, 2 mL/min).

### 3.5. Quantitative NMR Experiment

#### 3.5.1. Sample Preparation

Flos extracts (PLr and PLs, 10 mg each) and methyl 3,5-dinitrobenzoate (IC, 1 mg) were precisely weighed separately. Methyl 3,5-dinitrobenzoate (IC, 1 mg) was dissolved in 1 mL of DMSO-*d*_6_, and 150 μL of the IC solution was transferred to a glass vial containing 550 μL of DMSO-*d*_6_. Then, 650 μL of the resulting solution was added to 10 mg of the two extracts (PLs and PLr). Finally, 600 μL of the mixtures (IC with each extract) was transferred into a 5 mm NMR tube for the acquisition of the ^1^H NMR spectrum. Samples were prepared and analyzed in triplicate.

#### 3.5.2. NMR Data Acquisition and Processing

The NMR spectra were acquired in DMSO-*d*_6_ on a 600 MHz Bruker AVANCE NEO spectrometer (Oxford magnet, Switzerland) at the Core Research Support Center for Natural Products and Medical Materials (CRCNM). ^1^H NMR spectra were recorded using the following parameters: a calibrated 90° pulse (P1), relaxation delay (D1), 60.0 s; acquisition time (AQ), 4.0 s; number of scans (NS), 64; free induction decay (FID) data points, 256 K; spectral width, 17,857.1 Hz; receiver gain (RG), 32; temperature, 298 K. NMR spectra were processed and analyzed with Mnova 12.0.4 software (Santiago de Compostela, Spain). Post-acquisition processing was performed by zero-filling to 256 K, Lorentzian–Gaussian apodization (line broadening = −0.3, Gaussian factor = 0.05), manual phasing, and baseline correction (fifth-order polynomial). The residual DMSO-*d_6_* peak was referenced at 2.50 ppm. Global spectral deconvolution (GSD) was performed by performing peak picking of the entire spectrum and the GSD function in the MestReNova software with five fitting cycles [21]. This created a peak table containing all the deconvoluted peaks with their defining peak areas, which were assigned to the H-2 singlet signals at 8.2–8.5 ppm to quantify each isoflavone.

#### 3.5.3. Quantitative NMR Analysis

The purity of the seven isoflavonoids (**1**–**7**) was calculated using Equation (2), which is given below:

(2) $$P\left[\%\right]=\frac{n_{\mathrm{IC}}\cdot {\mathrm{Int}}_{t}\cdot {\mathrm{MW}}_{t}\cdot m_{\mathrm{IC}}}{n_{t}\cdot {\mathrm{Int}}_{\mathrm{IC}}\cdot {\mathrm{MW}}_{\mathrm{IC}}\cdot m_{s}}\times {\;P}_{\mathrm{IC}}$$

where P, n, Int, MW, t, m, IC, and s are the purity, number of protons, integral value, molecular weight, target analyte, mass, internal calibrant, and sample, respectively.

### 3.6. HPLC Analysis

HPLC analysis was performed on a Waters (Waters Corporation, Milford, CT, USA) Alliance 2695 separation module (Waters Co., Milford, MA, USA) using a photodiode array detector (Waters 996). Water (solvent A) and acetonitrile (solvent B) were used as the mobile phase at a flow rate of 1 mL/min and injection volume was 20 μL. The UV chromatograms were extracted at 254 nm wavelength for quantitative analysis. The gradient program was used: 0–25 min, 5–50% B; 25–26 min, 50–100% B; 26–37 min, 100% B; 37–38 min, 100–5% B; 38–50 min, 5% B. The injected sample concentration was 1.5 mg/mL in HPLC-grade MeOH. To prepare the stock solution, accurately weighed 1 mg of each of the three standards [tectorigenin (**2**), tectoridin (**5**), and tectorigenin-7-*O*-*β*-d-xylopyranosyl-(1-6)-*O*-*β*-d-glucopyranoside (**7**)] was dissolved in 1 mL of CH_3_CN:H_2_O (1:1), 100% MeOH, and 100% EtOH, respectively. Six concentrations each for compounds **2** and **5**; 250, 125, 62.50, 31.25, 15.62, and 7.81 μg/mL were injected to prepare the calibration curves: **2** (Y = 3 × 10^−5^X + 1.0257, R^2^ = 0.9997) and **5** (Y = 3 × 10^−5^X + 0.0895, R^2^ = 0.9998). The calibration curve was prepared with 300, 250, 125, 62.50, 31.25, and 15.62 μg/mL (six concentrations) for **7** (Y = 4 × 10^−5^X + 2.3828, R^2^ = 0.9997) (Figure S18). Each concentration of the standard was injected three times. Three replicates of PLs and PLr extracts were weighed (1.5 mg), dissolved, and vortexed in 1 mL of 100% HPLC MeOH. For standard samples, each prepared extract was injected three times.

## 4. Conclusions

In this study, the qHNMR method was developed and successfully applied as a simple, rapid, sensitive, and reliable tool to quantify all seven isoflavones in the Puerariae Flos extracts obtained by two different methods. The proton signals of quantified isoflavones from H-2 singlets of C-ring at 8.2–8.5 ppm were used for quantification because they are distinct and do not overlap with each other or with the signals from other impurities. Our qHNMR quantification method was validated by the conventional HPLC method, and the quantification results of qHNMR closely corresponded to those of HPLC. This is the first report on a validated qHNMR method to quantify the secondary metabolites of Pueraria Flos and to compare the data with those procured by the conventional HPLC method. Hence, this qHNMR method can facilitate the quality control of Puerariae Flos for the preparation and botanical standardization of medicinal products, while overcoming all the limitations of HPLC. Furthermore, this study can be a guideline for establishing quantitative and chemical fingerprinting methods using NMR for various natural products containing isoflavonoids as the main components.

## Figures and Tables

**Table 1:** Comparative results for isoflavone content in the extract of the flower of *P. lobata* by qHNMR and HPLC methods. Samples were prepared and analyzed in triplicate (*n* = 3).

Compounds	% *w*/*w* (Mean ± Std Dev) of PLs		% *w*/*w* (Mean ± Std Dev) of PLr	
	qHNMR	HPLC-UV	qHNMR	HPLC-UV
5-Methoxydaidzein (**1**)	0.95 ± 0.03	-	0.92 ± 0.01	-
Tectorigenin (**2**)	0.67 ± 0.01	0.65 ± 0.00	0.59 ± 0.02	0.49 ± 0.01
Genistin (**3**)	0.43 ± 0.02	-	0.53 ± 0.02	-
Glycitin (**4**)	1.96 ± 0.06	-	3.02 ± 0.05	-
Tectoridin (**5**)	1.86 ± 0.09	1.60 ± 0.01	2.42 ± 0.04	1.92 ± 0.02
7,4′-Dihydroxy-6-methoxyisoflavone 7-*O*-*β*-d-xylopyranosyl-(1-6)-*O*-*β*-d-glucopyranoside (**6**)	0.57 ± 0.03	-	0.98 ± 0.03	-
Tectorigenin-7-*O*-*β*-d-xylopyranosyl-(1-6)-*O*-*β*-d-glucopyranoside (**7**)	1.55 ± 0.07	1.32 ± 0.01	2.10 ± 0.05	1.75 ± 0.02
Total	7.99 ± 0.11		10.57 ± 0.10	

## Data Availability

All data generated or analyzed during this study are included in this published article.

## Supplementary Materials

The following supporting information can be downloaded at: https://www.mdpi.com/article/10.3390/plants11040548/s1, Figures S1–S7: ^1^H NMR spectra of compounds **1**–**7**; Figures S8–S14: LR-ESI-MS spectra of compounds **1**–**7**; Figure S15: Resonance at δ_H_ 8.45 ppm of tectoridin to determine LOD and LOQ using S/N ratio; Figure S16: The stacked ^1^H NMR spectra of the crude extract with IC before and after spiking of tectoridin; Figure S17: Accuracy determination using the recovery method; Figure S18: HPLC-UV profiles of the extracts (PLr and PLs) and calibration curves of tectorigenin (**2**), tectoridin (**5**), and tectorgenin-7-O-*β*-d-xylosylglucoside (**7**).
