# Supplementary material for: Absolute Quantification of Isoflavones in the Flowers of Pueraria lobata by qHNMR
Source: Plants (Basel). 2022 Feb 18;11(4):548. doi: 10.3390/plants11040548 (PMC8878989; doi:10.3390/plants11040548)

Supporting Information

Absolute Quantification of Isoflavones in the Flowers of *Pueraria lobata* by qHNMR

Punam Thapa [1], Hye Mi Kim [2], Joon-Pyo Hong [3], Ranhee Kim [3], Sunil Babu Paudel [1], Hyukjae Choi [1,4], Dae Sik Jang [2,3,*] and Joo-Won Nam [1,*]

[1] College of Pharmacy, Yeungnam University, Gyeongsan-si, Gyeongsangbukdo 38541, Republic of Korea; pansup35@gmail.com (P.T); phrsunil@gmail.com (S.B.P); h5choi@yu.ac.kr (H.C.); jwnam@yu.ac.kr (J.-W.N.)

[2] College of Pharmacy, Kyung Hee University, Seoul 02447, Republic of Korea; hyemi586@gmail.com (H.M.K); dsjang@khu.ac.kr (D.S.J.)

[3] Department of Life and Nanopharmaceutical Sciences, Graduate School, Kyung Hee University, Seoul 02447, Republic of Korea; ongjoon@naver.com (J.-P.H.); rhee0423@khu.ac.kr (R.K.); dsjang@khu.ac.kr (D.S.J.)

[4] Research Institute of Cell Culture, Yeungnam University, Gyeongsan, Gyeongbuk 38541, Republic of Korea; h5choi@yu.ac.kr (H.C.)

Figure	S1.	^{1}H NMR (600 MHz, DMSO-d_6) of 5-methoxydaidzein (**1**)...	3
Figure	S2.	^{1}H NMR (600 MHz, DMSO-d_6) of tectorigenin (**2**)..	4
Figure	S3.	^{1}H NMR (600 MHz, DMSO-d_6) of genistin (**3**)...	5
Figure	S4.	^{1}H NMR (600 MHz, DMSO-d_6) of glycitin (**4**)...	6
Figure	S5.	^{1}H NMR (600 MHz, DMSO-d_6) of tectoridin (**5**)..	7
Figure	S6.	^{1}H NMR (600 MHz, DMSO-d_6) of 7-*O*-*β*-D-xylopyranosyl-(1-6)-*O*-*β*-D-glucopyranoside (**6**)..	8
Figure	S7.	^{1}H NMR (600 MHz, DMSO-d_6) of tectorgenin-7-*O*-*β*- D-xylosylglucoside (**7**).................	9
Figure	S8.	LR-ESI-MS of 5-methoxydaidzein (**1**)..	10
Figure	S9.	LR-ESI-MS of tectorigenin (**2**)...	10
Figure	S10.	LR-ESI-MS of genistin (**3**)..	11
Figure	S11.	LR-ESI-MS of glycitin (**4**)...	11
Figure	S12.	LR-ESI-MS of tectoridin (**5**)..	12
Figure	S13.	LR-ESI-MS of 7-O-*β*-D-xylopyranosyl-(1-6)-*O*-*β*-D-glucopyranoside (**6**).........................	12
Figure	S14.	LR-ESI-MS of tectorgenin-7-O-*β*-D-xylosylglucoside (**7**)..	13
Figure	S15.	Resonance at δ_H 8.45 ppm of tectoridin to determine LOD and LOQ using S/N ratio..	14
Figure	S16.	The stacked ^{1}H NMR spectra of the crude extract with IC before and after spiking of tectoridin (Internal calibrant: methyl 3,5-dinitrobenzoate)..	15
Figure	S17.	Accuracy determination using the recovery method...	16
Figure	S18.	HPLC-UV profiles of the extracts (PLr and PLs) and calibration curves of tectorigenin (**2**), tectoridin (**5**) and tectorgenin-7-O-*β*-D-xylosylglucoside (**7**).....................................	17

Figure S1. ^{1}H NMR (600 MHz, DMSO-d_6) of 5-methoxydaidzein (**1**)

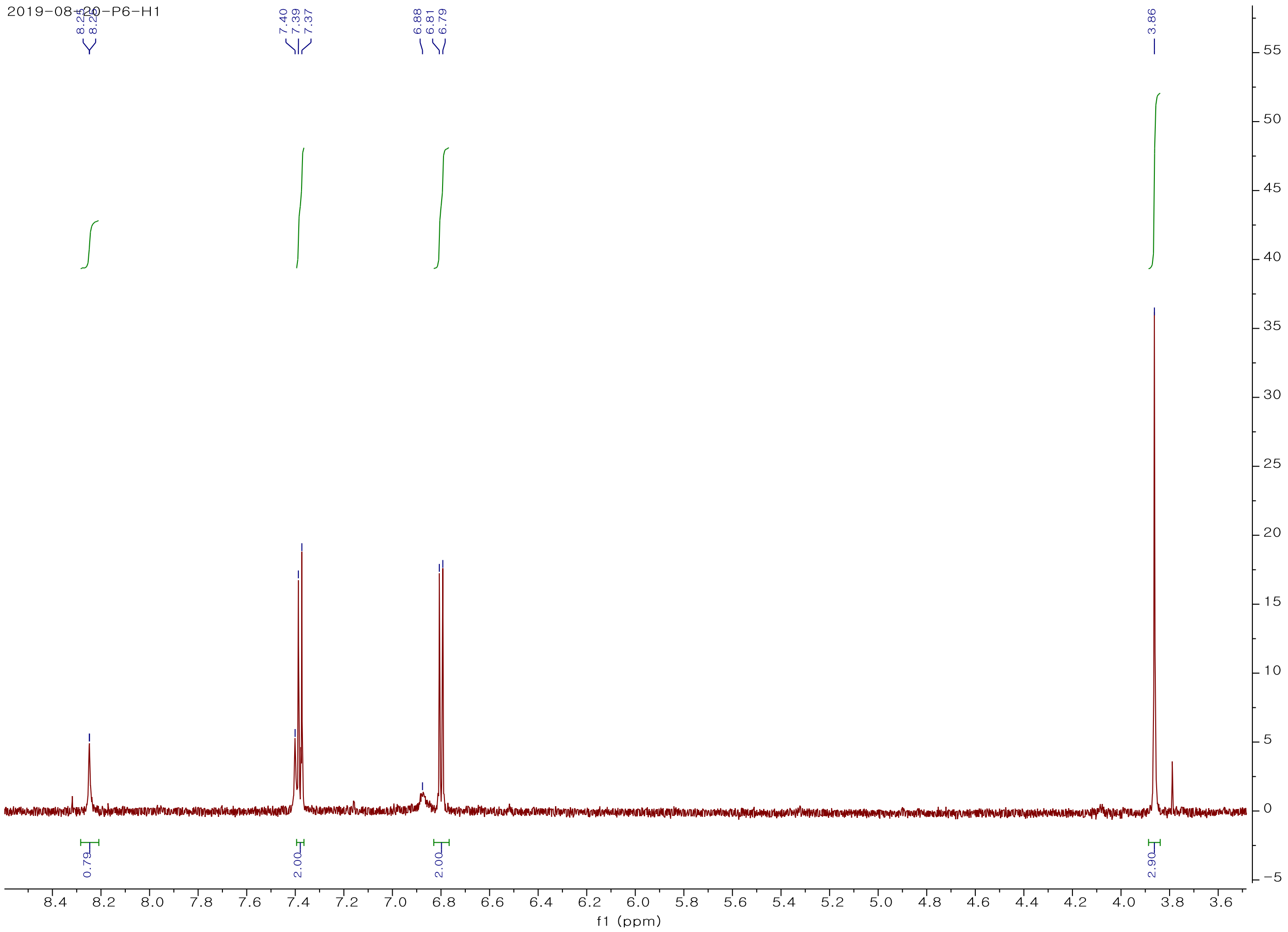

Figure S2. ^{1}H NMR (600 MHz, DMSO-d_6) of tectorigenin (**2**)

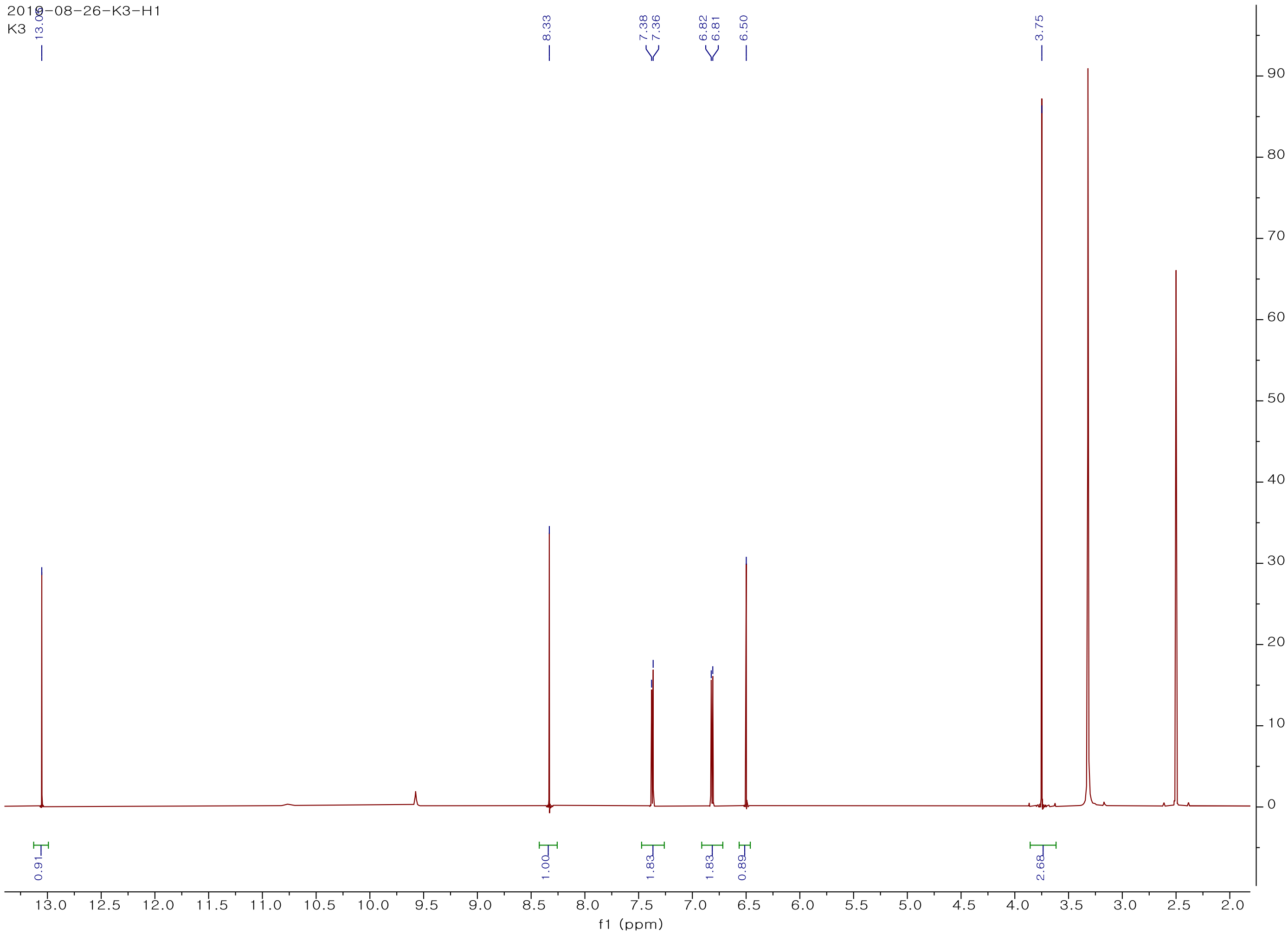

Figure S3. ^{1}H NMR (600 MHz, DMSO-d_6) of genistin (**3**)

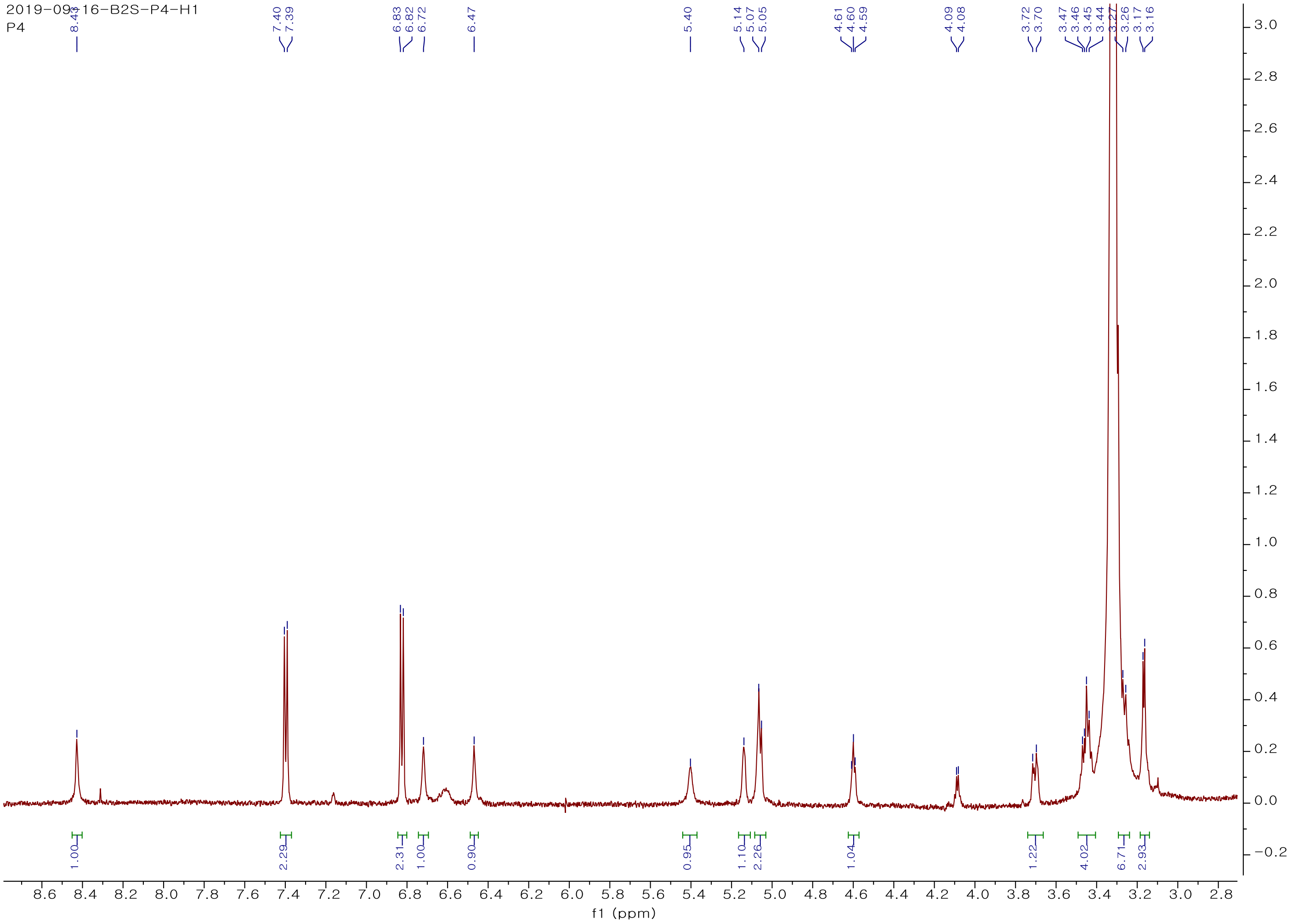

Figure S4. ^{1}H NMR (600 MHz, DMSO-d_6) of glycitin (**4**)

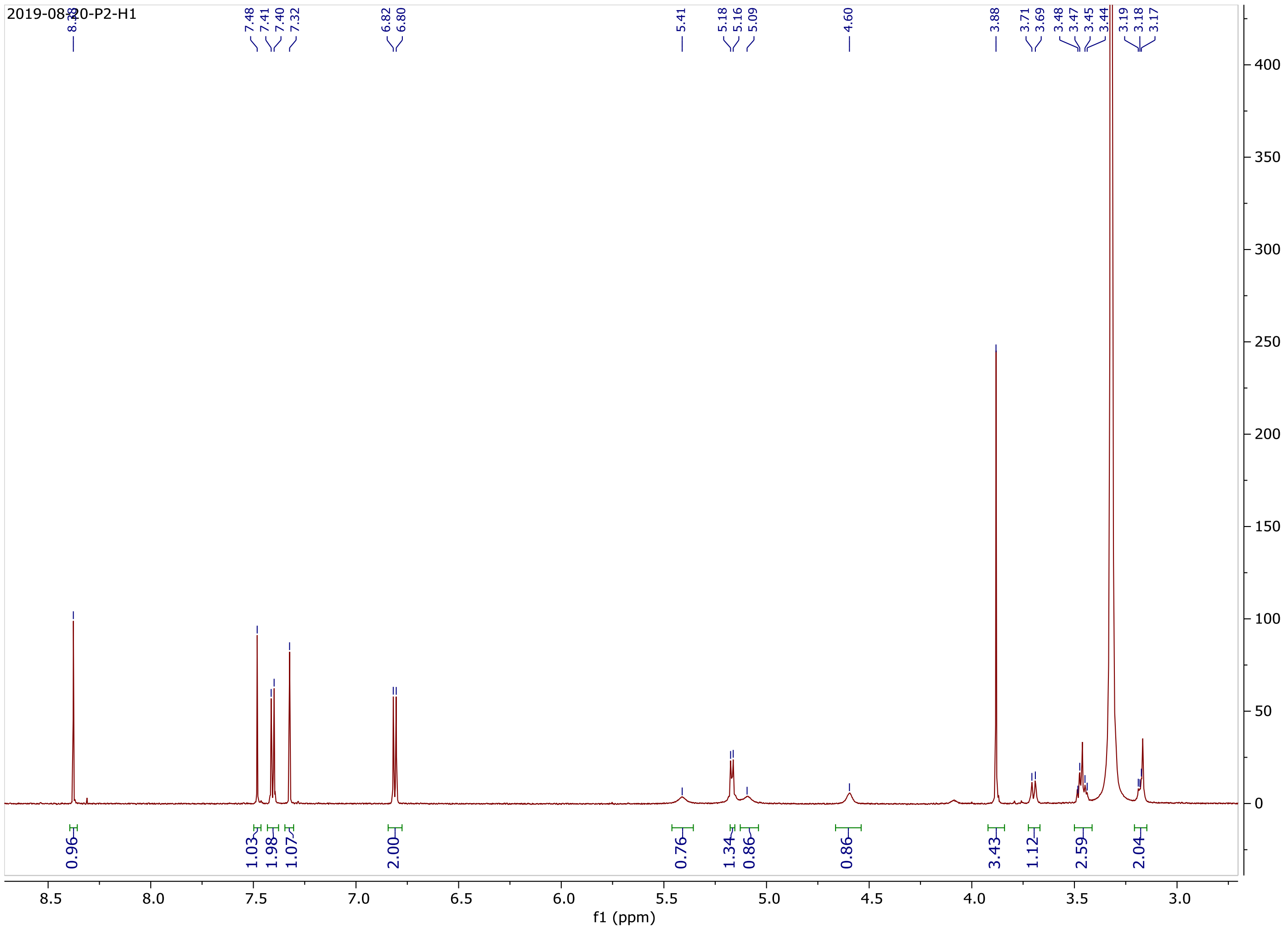

Figure S5. ^{1}H NMR (600 MHz, DMSO-d_6) of tectoridin (**5**)

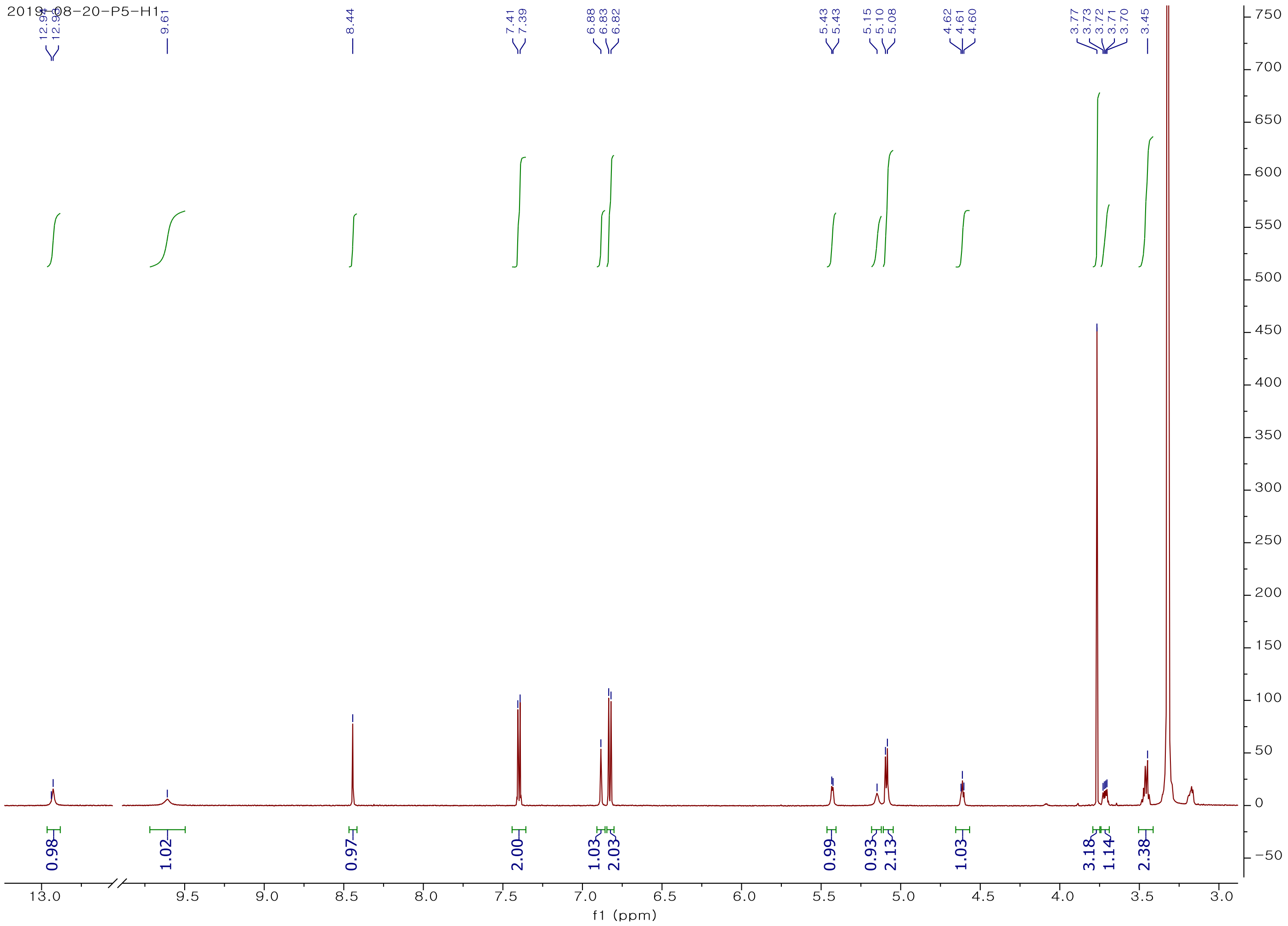

Figure S6. ^{1}H NMR (600 MHz, DMSO-d_6) of 7-*O*-*β*-D-xylopyranosyl-(1-6)-*O*-*β*-D-glucopyranoside (**6**)

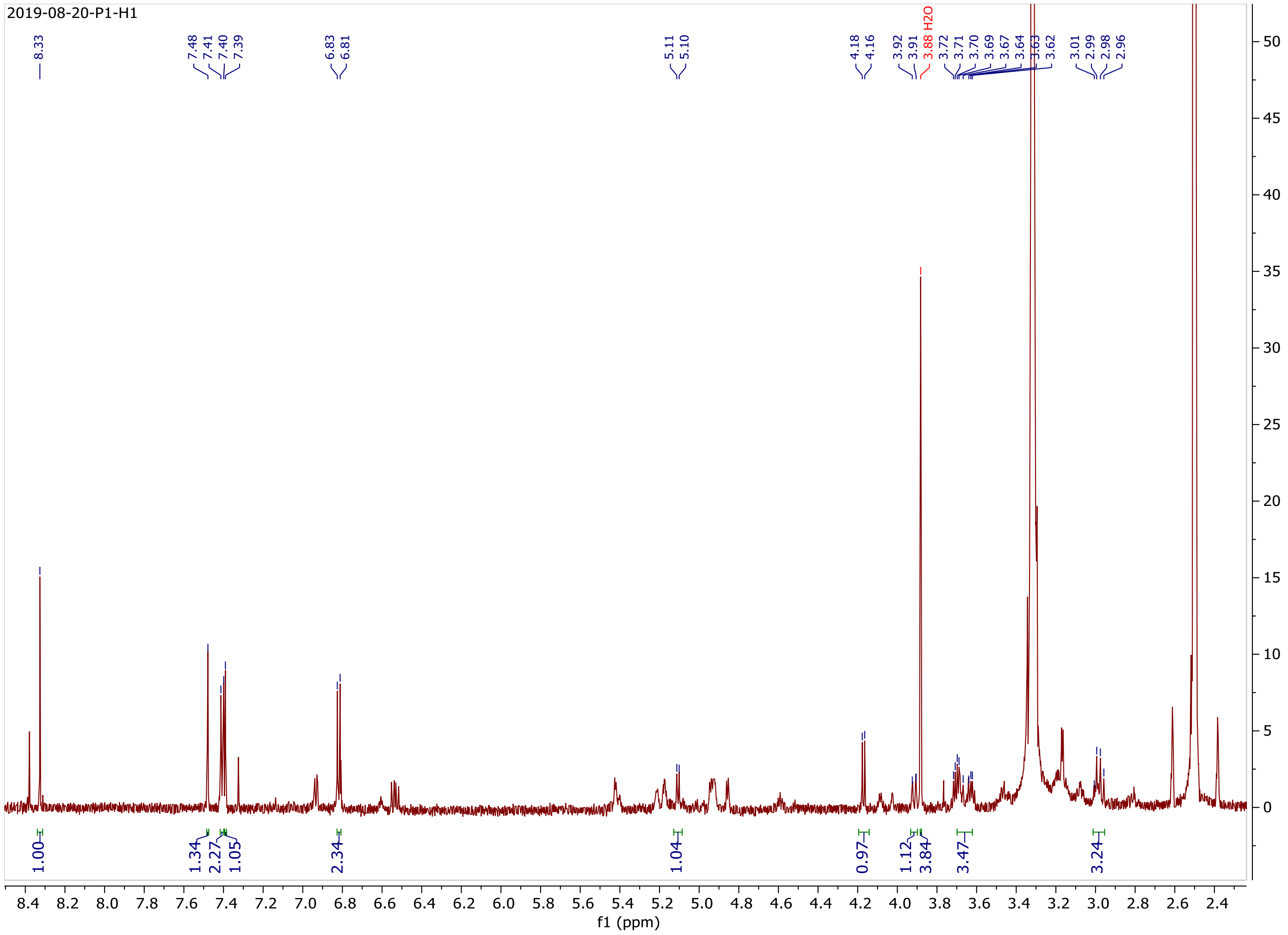

Figure S7. ^{1}H NMR (600 MHz, DMSO-d_6) of tectorgenin-7-*O*-*β*-D-xylosylglucoside (**7**)

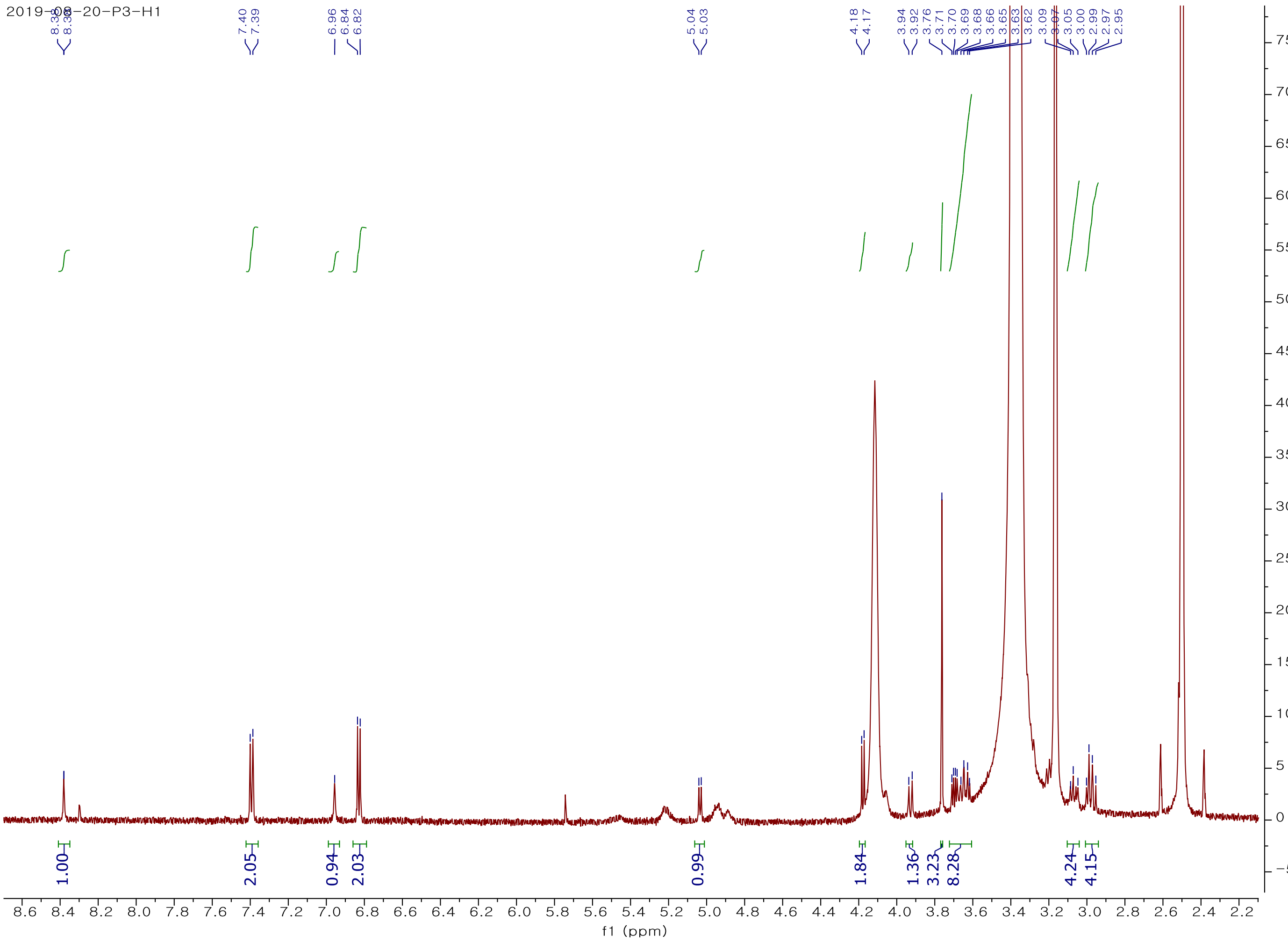

Figure S8. LR-ESI-MS of 5-methoxydaidzein (**1**)

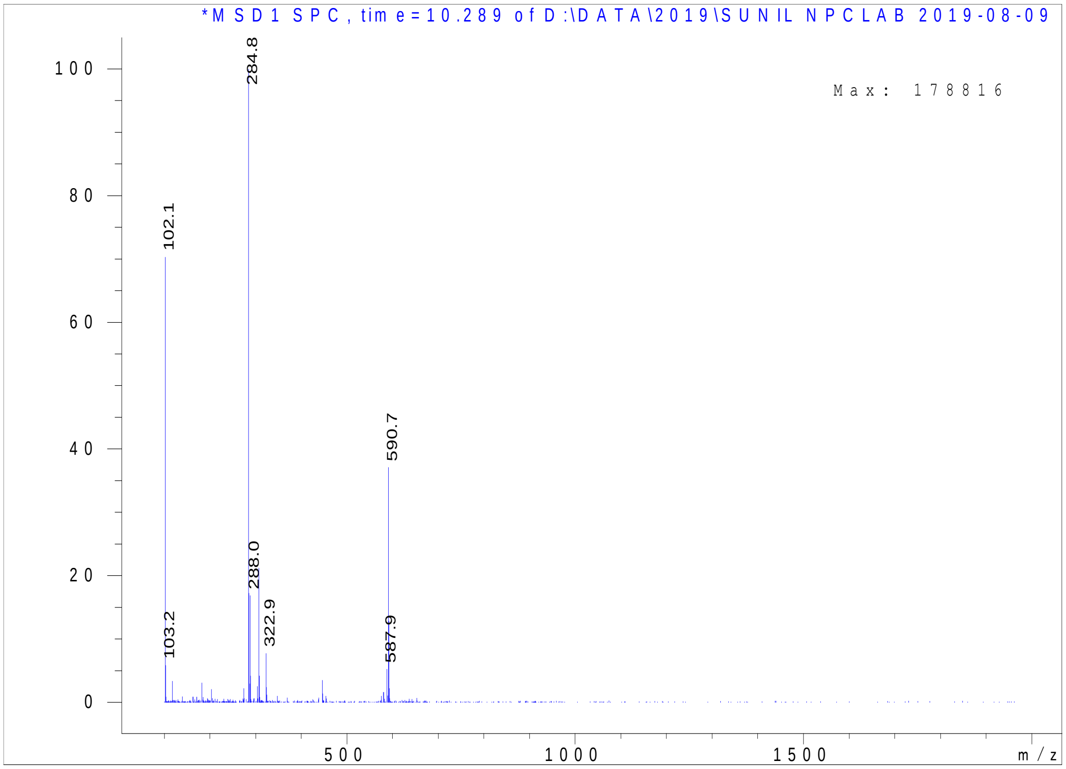

Figure S9. LR-ESI-MS of tectorigenin (**2**)

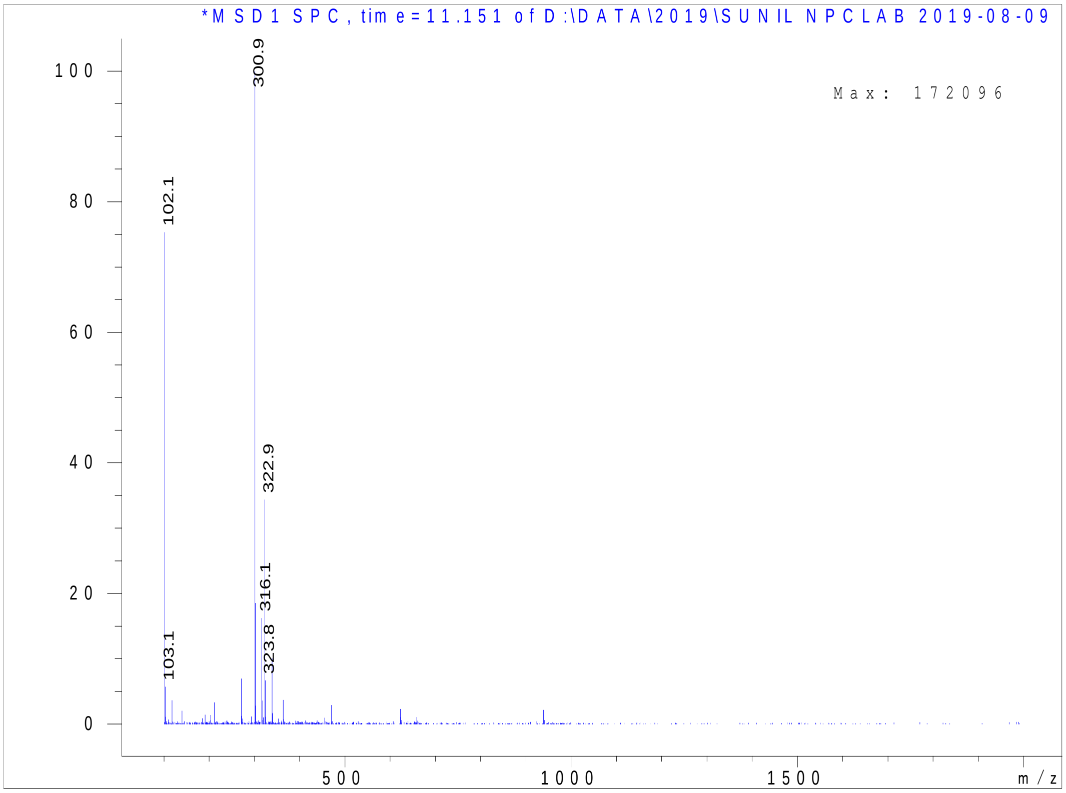

Figure S10. LR-ESI-MS of genistin (**3**)

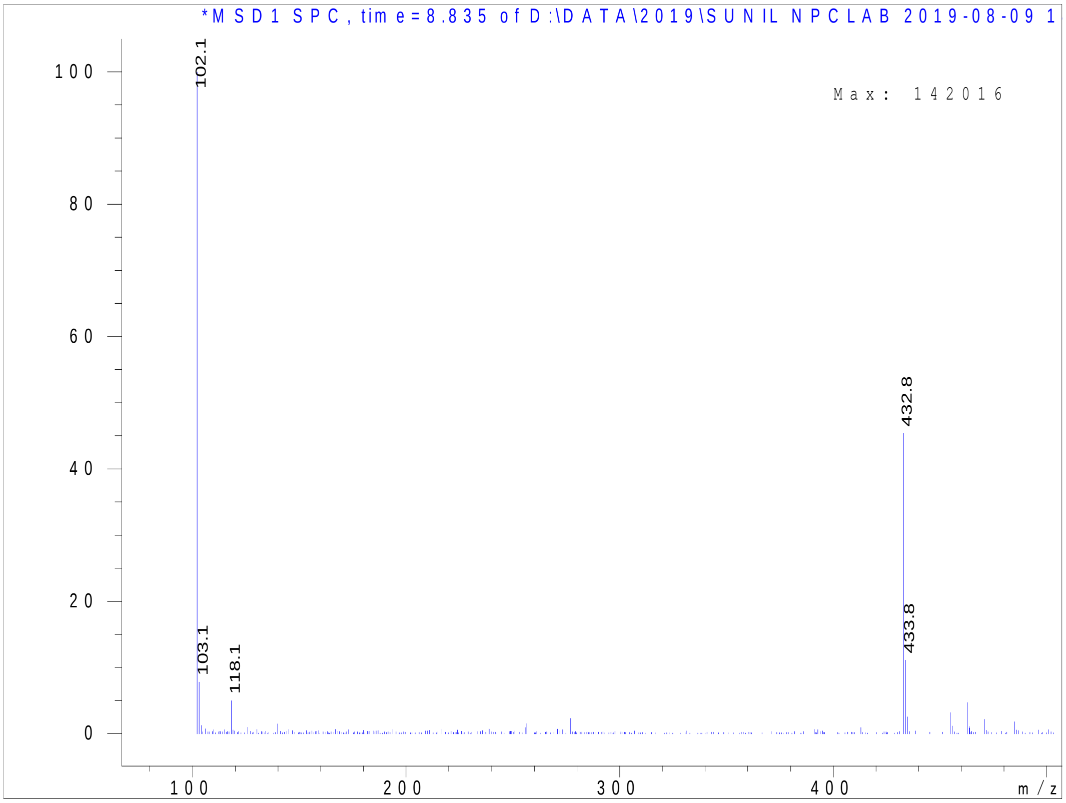

Figure S11. LR-ESI-MS of glycitin (**4**)

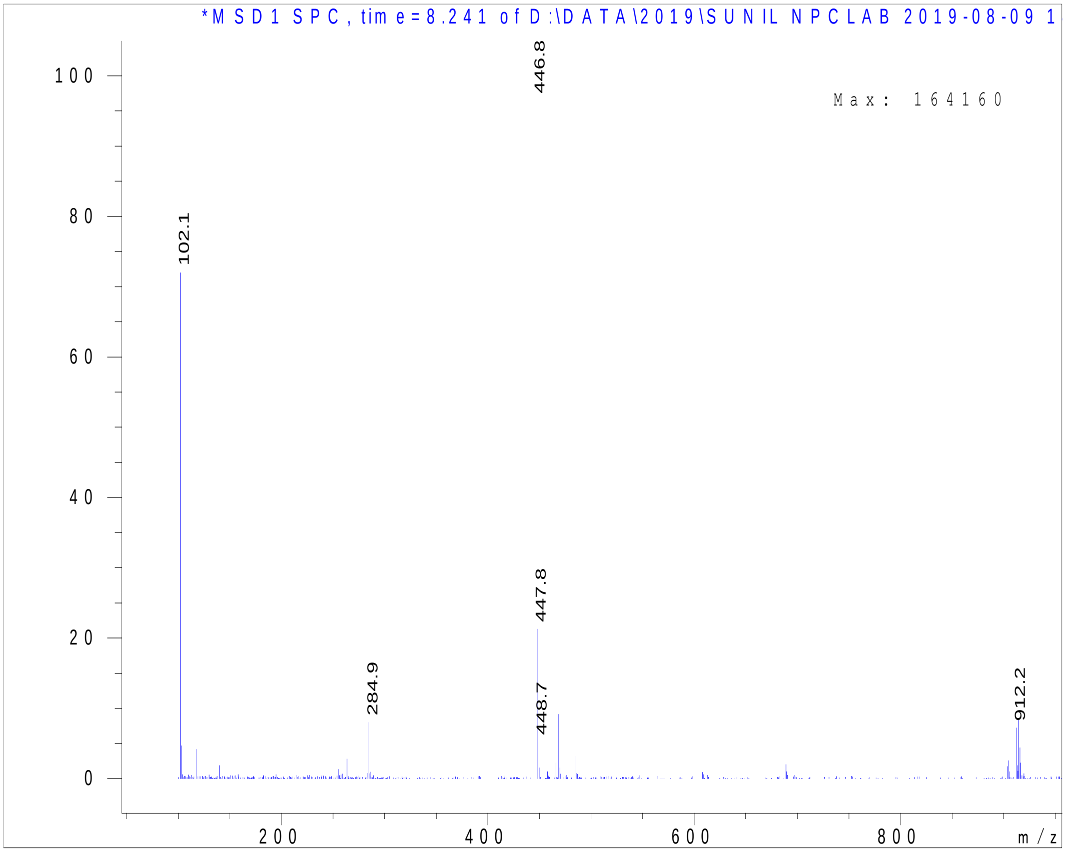

Figure S12. LR-ESI-MS of tectoridin (**5**)

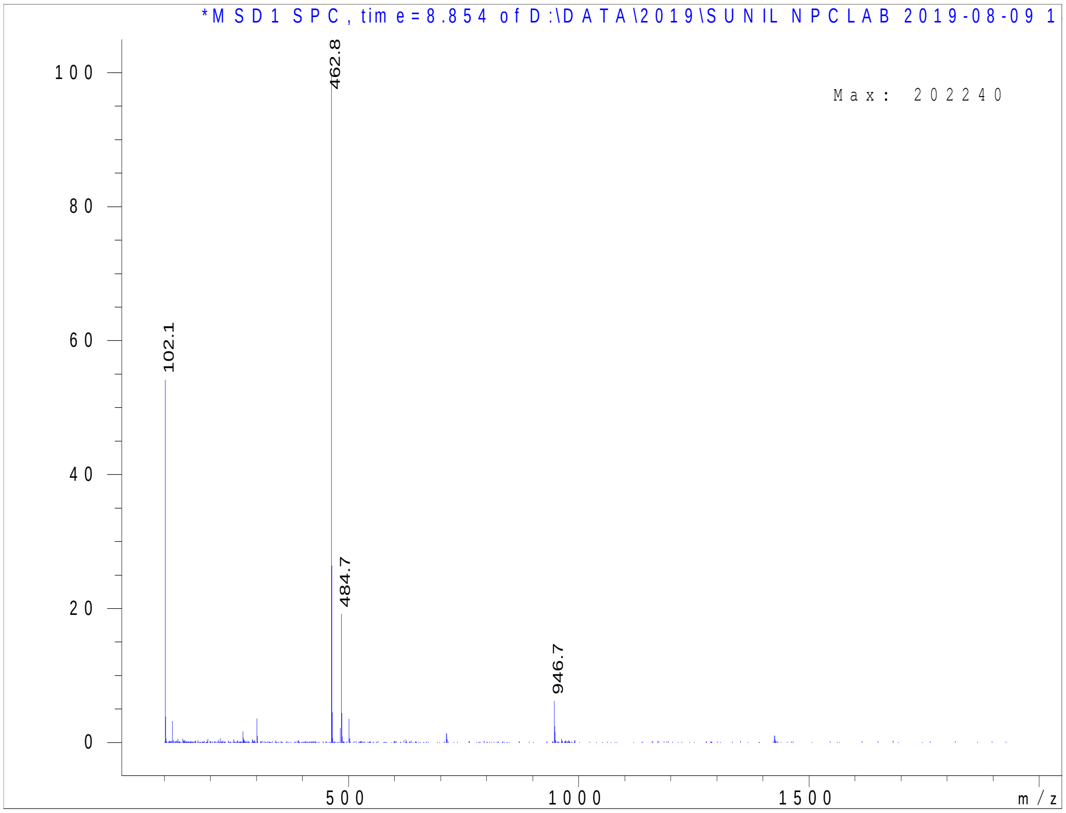

Figure S13. LR-ESI-MS of 7-O-β-D-xylopyranosyl-(1-6)-*O*-β-D-glucopyranoside (**6**)

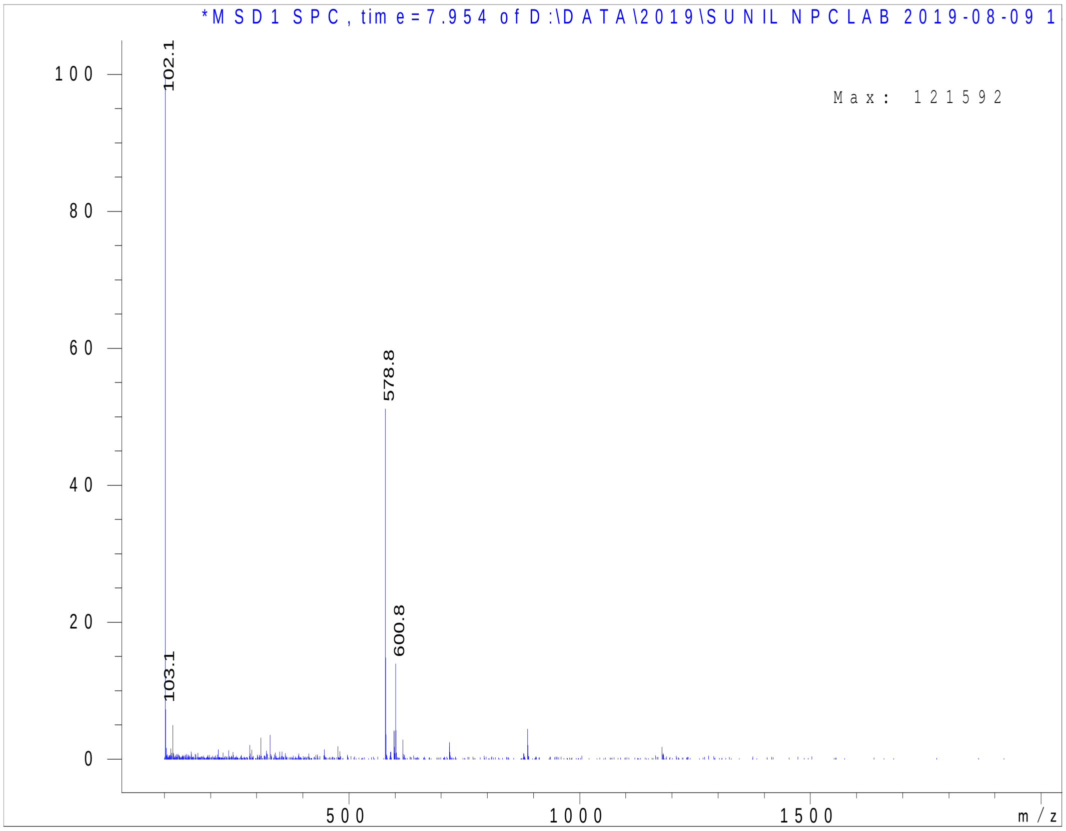

Figure S14. LR-ESI-MS of tectorgenin-7-O-β-D-xylosylglucoside (**7**)

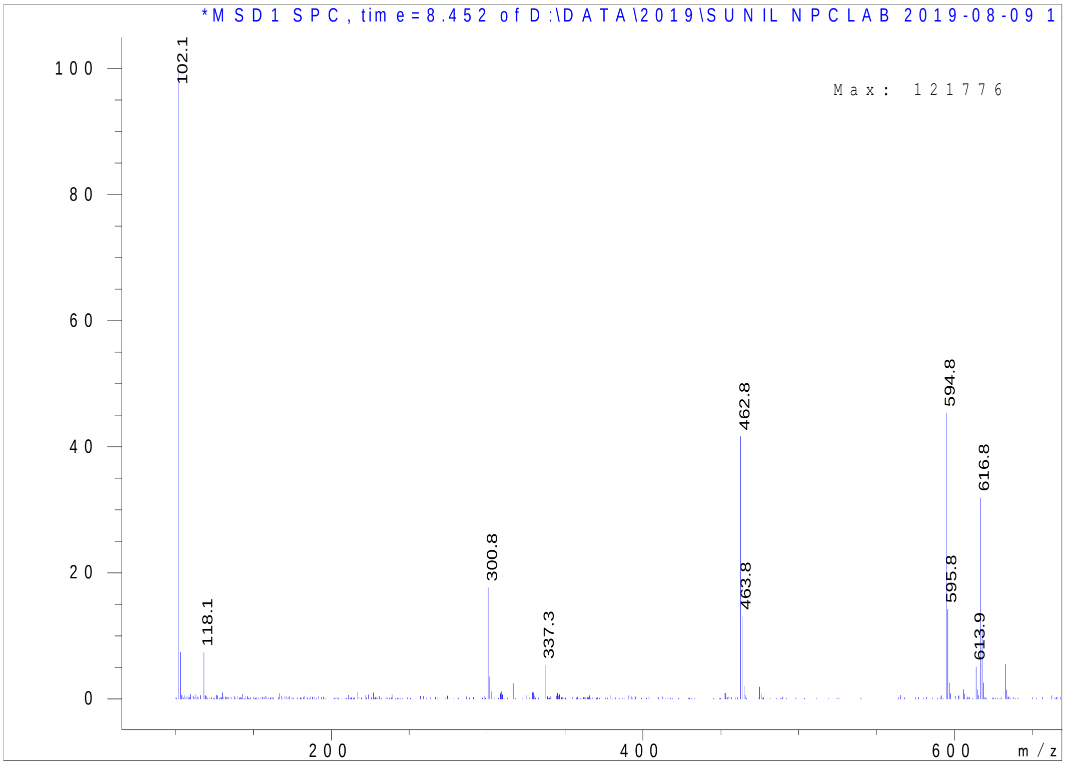

Figure S15. Resonance at δ_H 8.45 ppm of tectoridin to determine LOD and LOQ using S/N ratio.

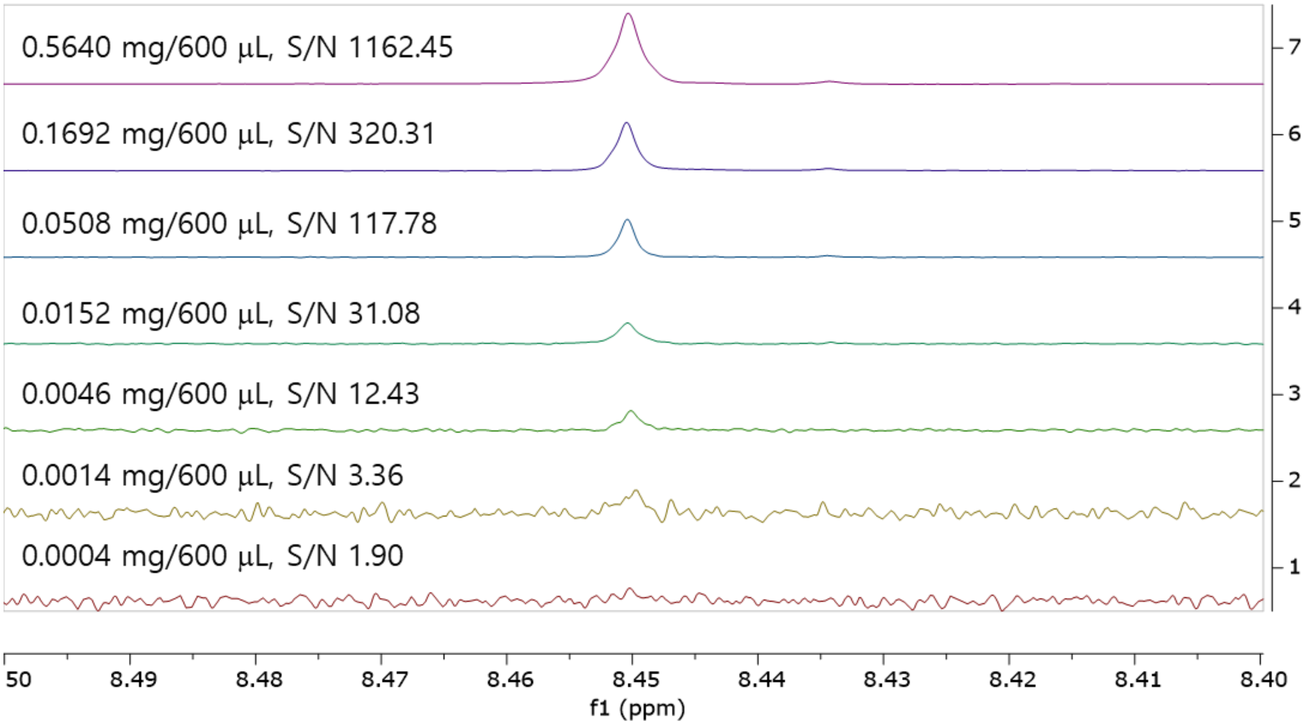

Figure S16. The stacked ^{1}H NMR spectra of the crude extract with IC before and after spiking of tectoridin (Internal calibrant: methyl 3,5-dinitrobenzoate).

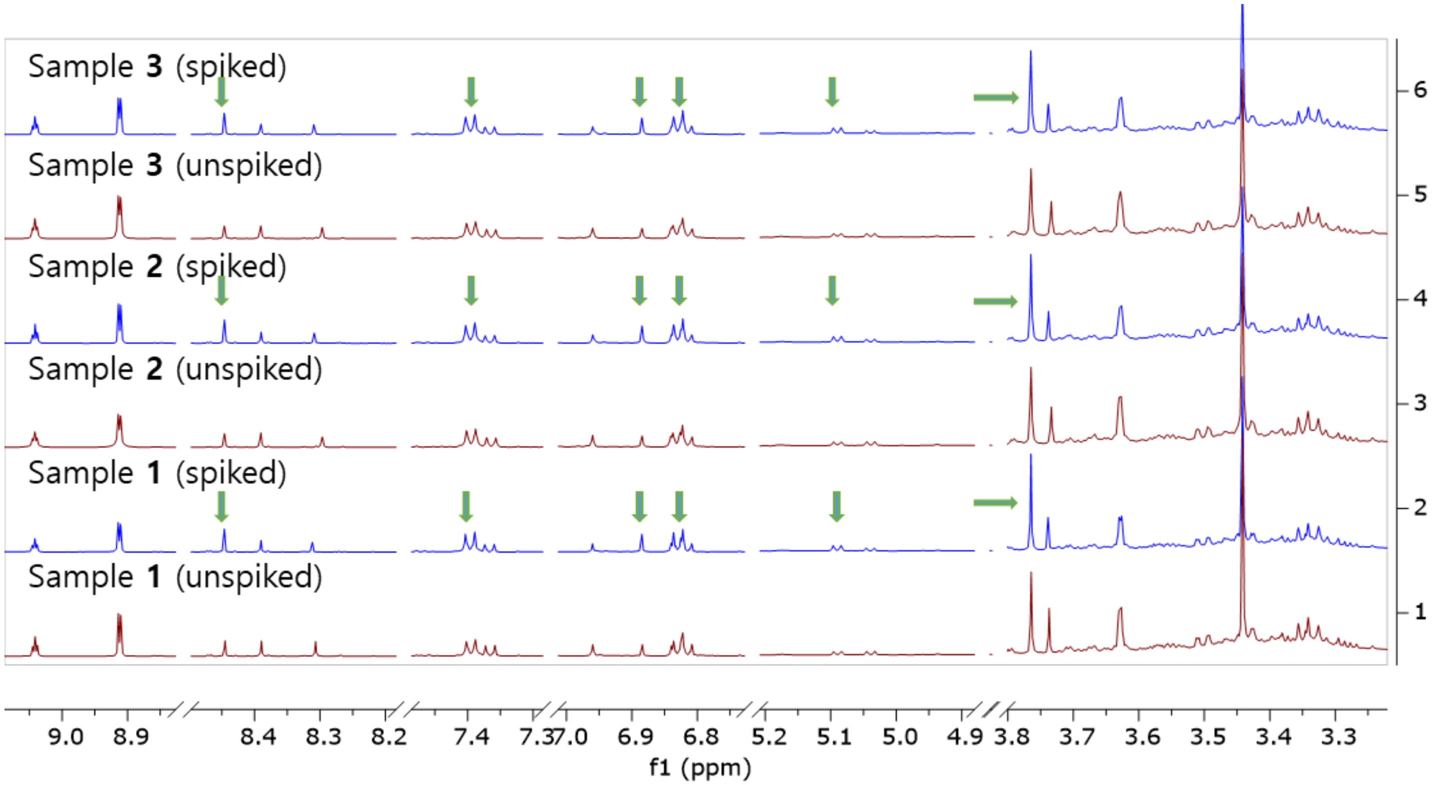

Figure S17. Accuracy determination using the recovery method

	Qty of tectoridin (mg)	% tectoridin after spiking	Qty of extract (mg)	Qty of tectoridin before spiking (mg)
Sample 1	1.2933	12.4000	10.43	0.6800
Sample 2	1.2976	12.9500	10.02	0.6753
Sample 3	1.2392	12.3800	10.01	0.6296
Qty of tectoridin spiked (mg)		0.6250		
**Accuracy**	true value (mg)	observed value (mg)	relative error (%)	recovery (%)
Sample 1	1.3050	1.2933	0.9059	98.1254
Sample 2	1.3003	1.2976	0.2125	99.5587
Sample 3	1.2546	1.2392	1.2420	97.5374
**Average**			0.7868	98.4072

Figure S18. HPLC-UV profiles of the extracts (PLr and PLs) and calibration curves of tectorigenin (**2**), tectoridin (**5**) and tectorgenin-7-*O*-*β*-D-xylosylglucoside (**7**).

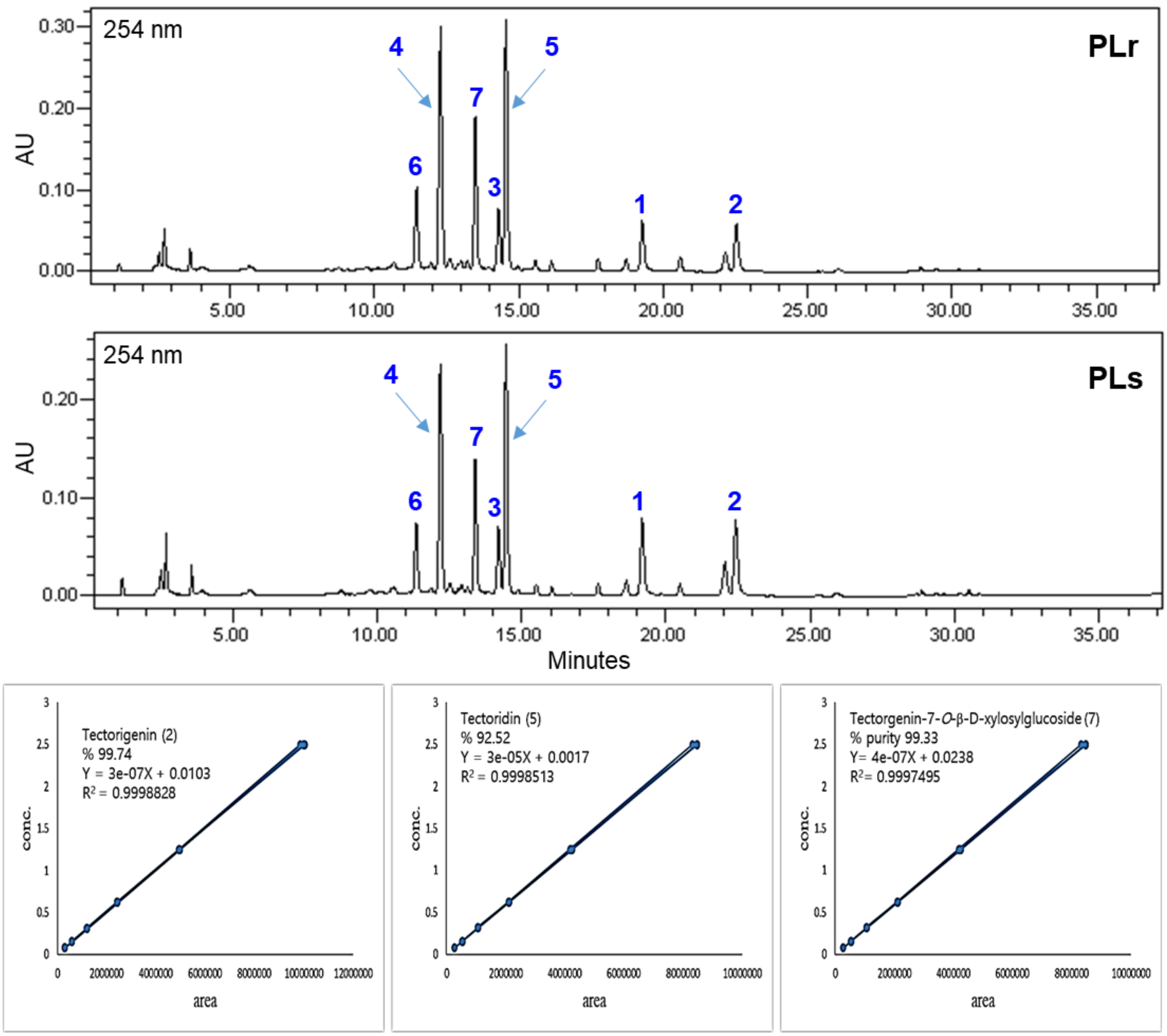

Supplement: Supplementary file 1 [file plants-11-00548-s001.zip › plants-1594001-supplementary.pdf]
